# Characterizing Pairwise U-Turn Behavior in Fish: A Data-Driven Analysis

**DOI:** 10.3390/e25121639

**Published:** 2023-12-09

**Authors:** Yuan Tao, Yuchen Zhou, Zhicheng Zheng, Xiaokang Lei, Xingguang Peng

**Affiliations:** 1 School of Marine Science and Technology, Northwestern Polytechnical University, Xi’an 710072, China; 2 College of Information and Control Engineering, Xi’an University of Architecture and Technology, Xi’an 710055, China

**Keywords:** rummy-nose tetra, fish interaction, time-series clustering, data analysis

## Abstract

We applied the time-series clustering method to analyze the trajectory data of rummy-nose tetra (*Hemigrammus rhodostomus*), with a particular focus on their spontaneous paired turning behavior. Firstly, an automated U-turn maneuver identification method was proposed to extract turning behaviors from the open trajectory data of two fish swimming in an annular tank. We revealed two distinct ways of pairwise U-turn swimming, named *dominated turn* and *non-dominated turn*. Upon comparison, the *dominated turn* is smoother and more efficient, with a fixed leader–follower relationship, i.e., the leader dominates the turning process. Because these two distinct ways corresponded to different patterns of turning feature parameters over time, we incorporated the Toeplitz inverse covariance-based clustering (TICC) method to gain deeper insights into this process. Pairwise turning behavior was decomposed into some elemental state compositions. Specifically, we found that the main influencing factor for a spontaneous U-turn is collision avoidance with the wall. In *dominated turn*, when inter-individual distances were appropriate, fish adjusted their positions and movement directions to achieve turning. Conversely, in closely spaced *non-dominated turn*, various factors such as changes in distance, velocity, and movement direction resulted in more complex behaviors. The purpose of our study is to integrate common location-based analysis methods with time-series clustering methods to analyze biological behavioral data. The study provides valuable insights into the U-turn behavior, motion characteristics, and decision factors of rummy-nose tetra during pairwise swimming. Additionally, the study extends the analysis of fish interaction features through the application of time-series clustering methods, offering a fresh perspective for the analysis of biological collective data.

## 1. Introduction

In a diverse ecological context, biological collective behaviors are commonly observed. Therefore, many studies involve tracking and analyzing the behaviors of these biological groups to investigate their interaction patterns, subsequently establishing models to deepen our understanding of the mechanisms underlying these behaviors. Researchers have modeled the self-organizing phenomenon of collective escape behavior in pigeon flocks, investigating how they spontaneously coordinate their actions when confronted with potential threats to achieve group escape [1]. In addition, the analysis of fish-school behavior data provided a deeper understanding of the interactions and coordination within the fish school and how to construct mathematical models based on behavioral data to describe the collective movement of fish schools was discussed in [2]. In studying starlings’ highly coordinated group flight movements, researchers sought to comprehend the mechanisms underlying these collective behaviors and developed a mathematical model to simulate this phenomenon [3].

Previous research has focused on spatial relationships among study subjects. However, in biological motion, time information also significantly influences decision-making. For example, to investigate the influence of a specific bird species’ behavior on its companion group and the entire flock, researchers assessed the temporal relationship between the flight directions of the studied bird and those of other birds [4]. By calculating the directional time delays between pairs of birds, the mutual influence relationships within the bird flock were determined. In addition to perceiving the external environment through vision, fish respond to changes in the surrounding environment by sensing subtle water movement and pressure gradients through their lateral lines [5]. However, the water flow process generated by the motion of surrounding individuals reaching the central individual takes some time. Therefore, in addition to considering spatial factors, one must also consider temporal factors in the analysis of fish motion. In summary, relying solely on information from a single moment is insufficient for precisely analyzing mutual influences in biological motion. The absence of temporal factors may lead to certain limitations in the analysis results. Therefore, incorporating time-series data allows for a more comprehensive analysis of mutual influences in biological motion, better explaining biological behavior.

Time series represent sequences of variables over time, and analyzing them can reveal connections between study subjects at adjacent time points. This temporal information is crucial for comprehensively understanding biological motion and decision-making. The Toeplitz inverse covariance-based clustering (TICC) method [6] segments and clusters time-series data to analyze the system’s motion states. We aim to apply time clustering analysis to explore motion characteristics and decision factors in biological motion systems.

Fish exhibit high maneuverability and are easily identifiable, making them common subjects for analyzing collective behavior [7,8]. Among these behaviors, the spontaneous turning behavior of fish holds significant importance in studying information transmission and interactions during group movement [9]. Analyzing interactions at the individual level is relatively straightforward and enhances our understanding of self-organizational processes within the group [10,11]. Therefore, we have chosen to focus on the trajectory data of fish’s pairwise turning behavior for our research.

This study analyzed rummy-nose tetra swimming in annular tanks using an open dataset in [9]. We developed an automated method to identify and extract pairwise U-turn behaviors from the data. We identified two distinct types of U-turn behaviors based on the extracted features. We compared these pairwise U-turn behaviors as *dominated turn* and *non-dominated turn*. A fixed and dominated relationship exists in *dominated turn*, whereas it does not exist in *non-dominated turn*. The *dominated turn* exhibited shorter turn durations, closer inter-individual distances, and smoother turning processes.

Moreover, we investigated the steering process of rummy-nose tetra pairs from a state perspective using subsequence clustering on multivariate time series. We applied the Toeplitz inverse covariance-based clustering (TICC) method [6] to cluster the state parameter dataset derived from the fish swimming process into five clusters. By analyzing the composition of U-turn states, distinct patterns emerged for the two turning behaviors, indicating the presence of different turning processes. We elucidated the decision factors that influence different U-turn behaviors based on the observed U-turn state compositions. These factors include wall avoidance as the main factor of spontaneous turns, adjustment of the distance from the wall through directional changes when the inter-individual distance is appropriate, and a combination of behavioral factors leading to collision avoidance nearby, resulting in a more complex process.

In the following sections, we describe the analyzed public trajectory dataset, outline the methodology for extracting pairwise U-turn behaviors, and present the distinctive features of the two U-turn behaviors. Subsequently, we discuss the macroscopic characteristics of the two steering behaviors and quantitatively analyze the data characteristics of the two steering maneuvers. We apply the time-series clustering method to cluster the pairwise swimming data and interpret the clustering results. Finally, we analyze the factors that underlie different U-turn behaviors based on the compositions of the two U-turn processes.

## 2. Results

### 2.1. Collecting U-Turn Datasets from Videos

To study the pair-turning behavior of rummy-nose tetra, we used a publicly available dataset of pairs swimming in a ring tank [9]. This dataset was identified and collected using the IDTracker software [12] on the three-dimensional position coordinates of individual motion processes, with a collection frequency of 50 frames. The total duration of the data we analyzed was 2.6 h. During the analysis, the height change of the individual was ignored, only the motion of the two-dimensional plane was considered, and the original position data were filtered to remove error points and measurement noise (see Section 3.1). Two fishes swam freely in an annular tank during the experiment and turned spontaneously. In this study, we focus on U-turn behavior, i.e., the swimming direction of the fish switching between clockwise and counterclockwise.

**Figure 1:**
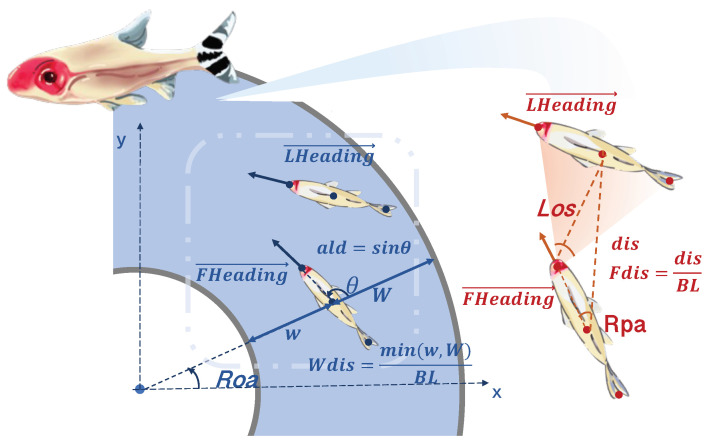
Geometric demonstration of the state parameters. The meanings represented by the parameters in the figure are as follows: BL represents the average body length of the fish. $\vec{LHeading(t)}$ and $\vec{FHeading(t)}$, respectively, represent the motion directions of the leader and follower. θ is the angle between the heading and the vector from the center of the circular water tank to the individual’s position. ***ald*** is the sine value of θ, indicating the relationship between an individual’s motion direction and the orientation of the walls. *w* and *W* are the distances from an individual to the inner and outer walls, respectively. ***Wdis*** is the normalized shortest distance from an individual to the walls. dis is the actual distance between two individuals. ***Fdis*** is the normalized distance between two individuals. ***Rpa*** represents the leader’s relative orientation in the follower. ***Los*** represents the leading individual’s projection angle in the follower’s field of view. ***Roa*** is the angle between the vector from the center of the circular tank to the individual’s position and the positive direction of the x-axis. The definitions and calculation methods for each parameter can be found in “Section 3.3”.

To automatically extract the corresponding U-turn data segments from the trajectory data (drawn from the 2.6 h video), we propose a method for identifying U-turn maneuvers in fish swimming within an annular water tank. As detailed in Section 3.2, we calculate the position rotation angle (*Roa*, as shown in Figure 1) according to the trajectory data and detect the U-turn events. Usually, the fish’s *Roa* decreases continuously when swimming clockwise and increases when swimming counterclockwise. When the U-turn occurs, the *Roa* curves exhibit a ‘∨’ or ‘∧’ shape, allowing us to identify the turning events by analyzing the extreme values of *Roa*. Since this identification method relies on a significant change in the *Roa*, i.e., a ‘∨’ or ‘∧’ shape, minor position deviations caused by measurements or small turning maneuvers do not result in false identifications. As a result, the method achieves an accuracy rate of 96.97% in identifying U-turn maneuvers (see table in Section 3.2). We only focus on single U-turn maneuvers where the exhibited leader–follower relationship is relatively fixed. This gives us a clear insight and understanding of pairwise U-turn behavior.

### 2.2. Two Pairwise U-Turn Behaviors

To further understand the pairwise U-turn behavior, we define one fish of the pairwise fish group as the leader if it is moving ahead in the direction of the group movement, and the other as the follower. For example, if the group moves counterclockwise, a fish is the leader if its *Roa* is larger than the other. Similarly, a fish is seen as the leader if its *Roa* is smaller than the other in a clockwise swimming case.

**Figure 2:**
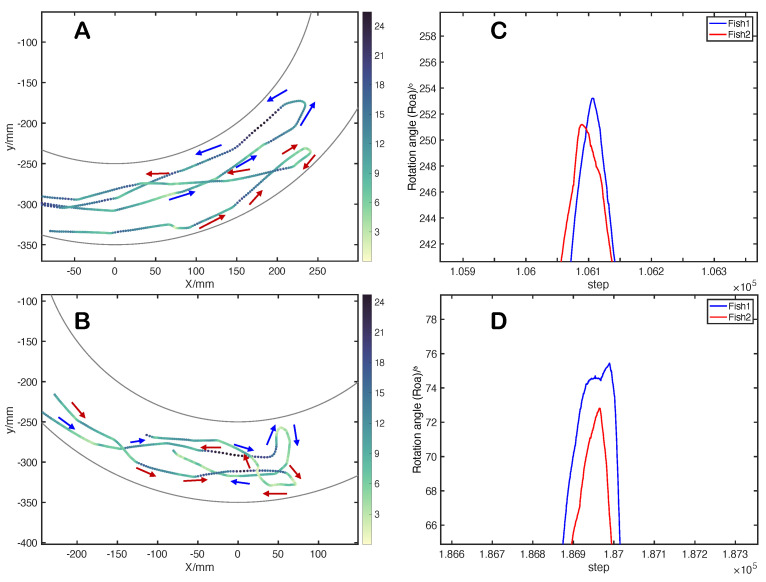
(**A**,**B**) represent the trajectory of *dominated turn* and *non-dominated turn*, respectively. The color bar indicates the changing rate of the Roa. The blue and red arrows in the figure represent the movement directions of Fish1 and Fish2. (**C**,**D**) represent the Roa curves corresponding to (**A**,**B**), respectively. Note that the Epoints (i.e., pairwise U-turn moment; see Section 3.2) of the pairwise U-turn process are 104,912 and 186,981 in the original dataset, respectively.

By analyzing the moving status of the fishes according to the U-turn dataset, we found two distinct pairwise U-turn behaviors. As for the first pairwise U-turn behavior, as shown in Figure 2C, the *Roa* curves of both fishes exhibit a ‘∧’ shape. Fish2 is the leader since it has a higher *Roa* value in the context of counterclockwise pairwise swimming and a lower *Roa* value in the clockwise pairwise stage. Fish1 follows the leader with a high alignment since the *Roa* curves are approximately parallel. The peak of Fish2’s ‘∧’ shape comes earlier than that of Fish1, indicating that Fish2 leads the pairwise U-turn process.

As for the second pairwise U-turn behavior, the leadership switches before and after the turning moment. In particular, Fish1 is the leader (with larger *Roa* when swimming counterclockwise) before the turning moment and turns earlier than the follower (Fish2). As shown in Figure 2D, when conducting the pairwise U-turn, the leader’s adjusting time is longer than in the first pairwise U-turn behavior. The changing trend in the leader’s *Roa* is relatively more gradual than that of Fish2 in Figure 2C. The pairwise U-turn process in Figure 2C is more complex because the leadership has been handed over from Fish1 to Fish2. The *Roa* value of Fish2 is smaller in the context of clockwise movement.

For consistent description, here we preliminary denote the first pairwise U-turn behavior as *dominated turn*, since the leadership is fixed, i.e., the follower is dominated by the leader, according to the above analysis and the motion analysis at the end of this section. In contrast, the second pairwise U-turn behavior is denoted as *non-dominated turn* since no fixed and dominated relationship is observed. Thanks to the proposed U-turn identification method, we collected 32 pairwise U-turn datasets, among which 14 were *dominated turns* and 18 *non-dominated turns*.

Figure 2A,B show the spatiotemporal characteristics of the *dominated turn* and *non-dominated turn*, respectively. In the *dominated turn*, two fishes maintain a certain distance and move in coordination before turning. During the pairwise U-turn process, Fish2 leads the deceleration and dominates the heading adjustment of both fishes. It is worth noting that during the paired U-turn process, the rotational angle curves of their positions are not entirely identical. The follower’s movement is not a complete replication of the leader; instead, the follower exhibits a faster deceleration rate and adjusts its heading. In *non-dominated turn*, there is no noticeable domination effect. The movements of both fishes are complex since they may “stop and go” at a relatively close distance. In addition, we introduce the definition of fish-school turning time from the literature [9]: the time from the moment when the first individual velocity direction is perpendicular to the outer wall in the group to the moment when the last individual velocity direction is perpendicular to the exterior wall. Specifically, the group turning time of *dominated turn* is 0.8496 s, and the group turning time of *non-dominated turn* is 1.6775 s. Therefore, we can conclude that the process of *dominated turn* is fast and smooth, while the maneuvering process of *non-dominated turn* has a lower speed and more stops.

The core action of the rummy-nose tetra turning process is a C-shaped turn [13,14]. In the fish’s C-shaped turn, the first half of the turn is information collection and acceleration, and the second half is uncontrolled gliding [2]. Since we are exploring the role of the decision-making elements, we only focus on the first half of the turning in the following data analysis. In particular, we statistically analyzed the datasets for the two U-turn maneuvers. The state variables we analyze include the relative distance between individuals (*Fdis*, the ratio of the distance between two individuals to their average body length), relative position angle (*Rpa*, representing the relative orientation of the leader to the follower), line-of-sight angle (*Los*, representing the projection angle of the leading individual in the follower’s field of view), and alignment degree [9] (*ald*, representing the relationship between the individual orientation and the wall, which is an essential indicator for judging the orientation of the individual). See Figure 1 for the geometric demonstration of the parameters and see “Section 3.3” for the formulation of each parameter.

**Figure 3:**
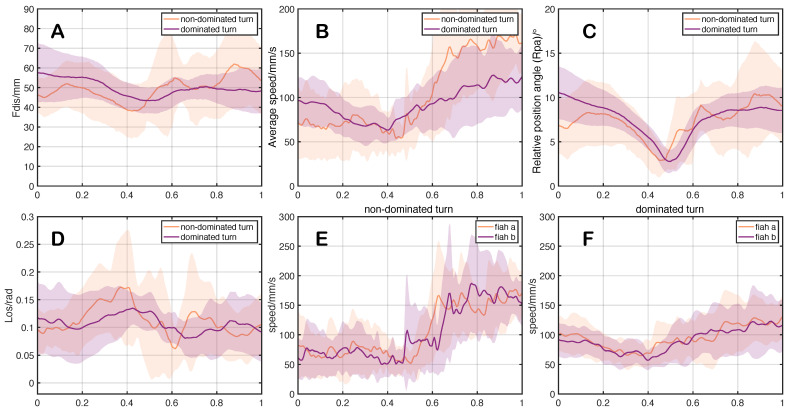
Curves of the state parameters for two types of pairwise U-turn processes. (**A**) represents the change curve of the distance (Fdis) between individuals in dominated turn and non-dominated turn; (**B**) represents the average speed change curve of two types of pairwise U-turn processes; (**C**) represents the change curve of relative position angle (Rpa) in two types of pairwise U-turn processes; (**D**) represents the change curve of Los in two types of pairwise U-turn processes; (**E**) represents the speed change curve of two individuals in non-dominated turn; (**F**) represents the speed change curve of two individuals in dominated turn. The horizontal axis in the graph represents the time from the beginning to the end of the turn. The turning duration has been standardized for comparative analysis to [0, 1].

It can be seen from Figure 3 that in the early stage of *dominated turn* in the pair, the distance between individuals is larger; the relative position angle is larger, which means that the leader is biased towards the follower’s lateral orientation; the *Los* changes relatively smoothly. When the pair perform a *non-dominated turn*, the distance between individuals is smaller; the leader is biased toward the follower’s forward orientation, so the relative position angle is smaller; the *Los* changes drastically. However, the speed difference between the two U-turn processes is not significant. Overall, the performance of the *dominated turn* is more stable and efficient.

### 2.3. Analyzing Paired U-Turn with Time-Series Clustering

While we have observed two distinctive pairwise U-turn behaviors and gained some analytical insights, elucidating the behavioral aspects of the turning process remains unresolved. Our subject of analysis has been time-series data of pairwise U-turns, and we have attempted to use time-series clustering methods to segment and interpret it.

A time series is a set of random variables arranged in chronological order. It is typically the result of observing a specific underlying process at equal intervals, based on a given sampling rate, revealing relationships between the past and the future. We considered two fish swimming in pairs as a system and calculated a parameter dataset from trajectory data that can describe the system’s state. We can uncover the inherent connections between parameters at adjacent moments by analyzing time-series data, essential for analyzing the pairwise U-turn decision-making process of fish.

We used the Toeplitz inverse covariance-based clustering (TICC) method [6] to segment and cluster the parameter data time series describing the pairing behavior of fish in the system. This method defines each cluster as a dependency network or Markov random field (MRF), where the network represents the topological relationships between parameters within the same state. This characterization captures the interdependencies among different parameters within the cluster, greatly enhancing the interpretability of the algorithm itself. We employed the TICC method to cluster the time-series dataset of the motion process into multiple clusters. Subsequently, by analyzing the node betweenness centrality scores and the corresponding motion state features within each cluster, we mapped each cluster to several states in the system’s motion process.

**Figure 4:**
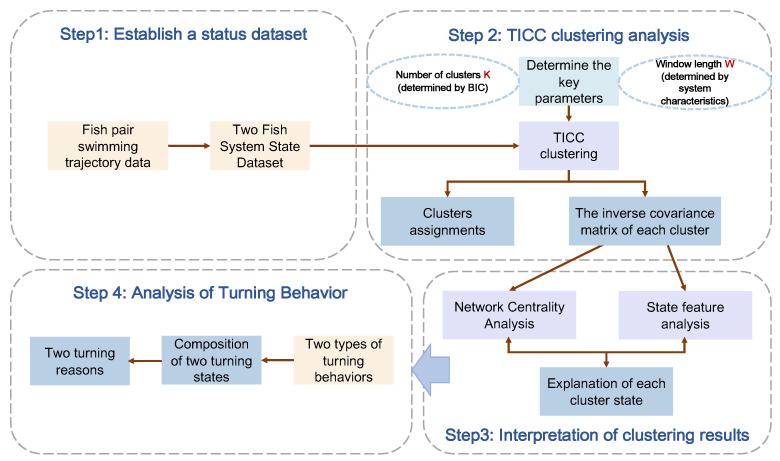
Diagram of time-series clustering of pairwise U-turn data. First of all, a state dataset was built describing fish pair swimming. Then, the key parameter values were determined, the TICC method was used for clustering, and each cluster’s cluster assignments and inverse covariance matrix were obtained. After that, the clustering results were interpreted by combining network centrality analysis and motion state data characteristics. Lastly, the state composition of the two turning maneuvers was analyzed and the reasons for the two turning maneuvers according to the state composition mode were explored.

Therefore, we subsequently aimed to decompose the turning process into interpretable state compositions by employing clustering and interpretation techniques. We tried to explain each state composition using the network centrality analysis technique, as shown in Figure 4.

First, we established a time series according to the trajectory data of the pairwise U-turn process. Within this time series, there were ten state parameters: (1) the distance between two fishes Fdis; (2) the shortest distance between the leader and walls $W_{l}dis$; (3) the shortest distance between the follower and walls $W_{f}dis$; (4) the relative position angle Rpa; (5) the leader’s alignment degree ald; (6) the follower’s alignment degree ald; (7) the leader’s speed Lspeed; (8) the follower’s speed Fspeed; (9) the leader’s heading $\vec{D_{l}Heading}$; and (10) the follower’s heading $\vec{D_{f}Heading}$. Those parameters are formulated in Section 3.3 and demonstrated in Figure 1.

Second, we used the Toeplitz inverse covariance-based clustering (TICC) [6] method to decompose the pairwise U-turn behavior into some state compositions. Using the TICC, we could cluster the time series into several state compositions with different network topologies (interdependence between state parameters). There are two algorithmic parameters in the TICC process: the number of clusters *k* and the window length *w*. We used k=5 according to the Bayesian information criterion (BIC) [15]. *w* corresponds to the maximum number of layers of the Markov network. According to our preliminary tests, the value of *w* in this study has good robustness within a specific range, and we let w=20.

**Figure 5:**
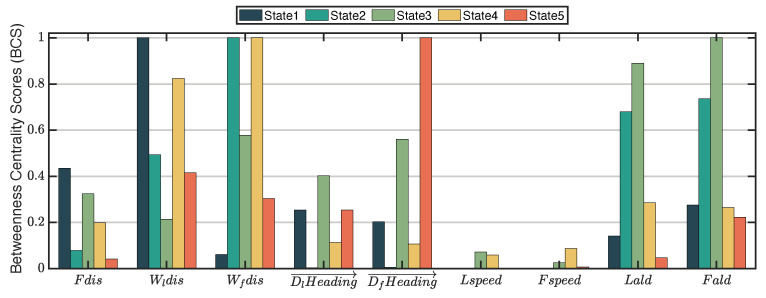
Ranking the betweenness centrality scores of state variables in each of the five states, the height of the bar graph in the figure represents the normalized score. A higher parameter ranking in the Table indicates a greater direct influence on other parameters and higher importance within the cluster. The definitions and data values of the parameters involved in the figure are detailed in in the table of Section 3.4.

**Figure 6:**
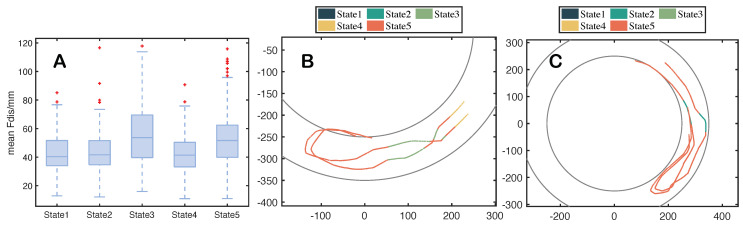
(**A**) Statistical chart of average Fdis in each state. The average Fdis refers to the mean distance among different data segments within that state. The box plots display the distribution of the average Fdis for the analyzed states. The lines in the box plots correspond to the median of the average Fdis for that state. The symbol ”+” represents data outliers. (**B**) *Dominated turn* trajectory with state compositions. (**C**) *Non-dominated turn* trajectory with state compositions. Note that the experimental device for collecting the dataset is a trapezoidal, circular water cylinder with a large and small upper cross-section. This is to avoid obstructing the cylinder wall when collecting the videos above. The two circles shown in the figure are at the bottom of the inner and outer walls, so the two-dimensional trajectory of the fish may intersect with the circles.

Third, we interpreted the clusters (i.e., the state compositions) obtained after the TICC process. In particular, the betweenness centrality score (BSC) [16,17] was utilized to calculate the relative “importance” of each node (state parameter) in the network. Therefore, we could rank the parameters’ BSC value in each state composition, as shown in Figure 5. The higher the ranking is, the more significant the impact of a parameter on others (numerical values are given in the table of Section 3.4). To better understand the five state compositions (k=5), we plot them in different colors, as shown in Figure 6. Thus, we can interpret the five state compositions as follows:

- **State 1**: The leader avoids the walls while the follower maintains its original movement trend. In this state, the distance between the leader and the wall exerts the most significant impact on the network, with the position adjustment parameters of the follower significantly influencing the behavior.
- **State 2**: In this state, two fishes actively avoid collisions with walls by adjusting the alignment (*ald*), resulting in a relatively organized overall process.
- **State 3**: The movement consistency between the leader and the follower is low, suggesting that the follower’s attention shifts during this stage.
- **State 4**: In this state, the collision avoidance effect between the two fish and the wall is achieved through the combined actions of multiple factors, including changes in the relative position and orientation between individuals, the relative position between individuals and the wall, and the motion state of each individual. As a result, the process becomes relatively complex.
- **State 5**: In this state, the follower replicates the leader’s movement by aligning its heading with the leader’s, resulting in motion coherence.

A comprehensive analysis of the five states determined that the distance factor significantly influences the pairwise U-turn process, while the relative orientation (Rpa) and the speeds (Fspeed and Lspeed) have relatively insignificant effects.

Finally, we analyzed the pairwise U-turn process at the behavioral level. As illustrated in Table 1, during the early stage of the *dominated turn*, the most prevailing behavior is characterized by state 5 transitioning to state 2. However, in the early stage of the *non-dominated turn*, the behavior is more intricate, but both state 3 and state 4 are present. Considering the interpretation of each state mentioned above, it can be inferred that the primary reason for the U-turn is the avoidance of wall obstacles. Notably, in the *dominated turn*, collision avoidance is accomplished by altering the direction of motion for both individuals. The collision avoidance process becomes more complex during the *non-dominated turn* as it involves comprehensive adjustments in the relative position and orientation of the individuals, their position relative to the wall, and the individual’s motion state. Additionally, there are instances where the follower’s attention changes the *non-dominated turn*. Analyzing the data characteristics of each state, we observed that maintaining an appropriate distance within the two-fish system promotes more orderly movement, thereby increasing the likelihood of a *dominated turn*. Conversely, when the distance between individuals is shorter, there is a higher probability of a *non-dominated turn* occurring.

## 3. Methods

### 3.1. Data Collection and Filtering

We collected a dataset of paired swimming trajectories of rummy-nose tetra fish in an experimental environment with a trapezoidal, circular water tank. The tank has a bottom inner diameter of 25 cm, an outer diameter of 35 cm, and a water depth of 7 cm. The dataset uses a Cartesian coordinate system with the center of the circular tank as the origin. The trapezoidal shape of the tank was designed to minimize interference from shadows during data collection, with the tank being wider at the top and narrower at the bottom. The experimental subjects were rummy-nose tetra, known for their strong schooling behavior, and they had an average body length of 33.3 mm. Note that we did not conduct experiments on live fish. The trajectory data of fish movement we used in this paper can be found in [9].

The dataset includes erroneous points and measurement noise, evident as “Z” shaped deviations on the trajectories, that do not represent actual fish movement. Such noise can substantially disrupt subsequent data processing and analysis. We employed the five-spot triple smoothing method on the raw position data to mitigate this issue. This method successfully filters out erroneous points and measurement noise while retaining the burst-gliding behavior characteristics of individual fish.

### 3.2. Extracting U-Turn Behavior

We determine whether an individual fish performs a U-turn based on the change in the position rotation angle during its swimming process. The position rotation angle (Roa), as demonstrated in Figure 1, is defined as the angle between the vector from the center of the circular tank to the individual’s position and the positive direction of the x-axis at the current moment.

(1) $$Roa\left(n,t\right)=\frac{\vec{l_{\left(n,t\right)}}\cdot \vec{x_{0}}}{\left|\vec{l_{\left(n,t\right)}}\right|\times 1}$$

Among them, Roa(n,t) is the position rotation angle of individual *n* in the fish school at time *t*, $\vec{l_{\left(n,t\right)}}$ is the vector whose center of circle points to the current position of individual *n* at time *t*, and $\vec{x_{\left(0\right)}}$ is the unit vector in the positive direction of the x-axis.

When the fish swims clockwise, the position rotation angle Roa decreases, while it increases when the fish swims counterclockwise. Moreover, there is a sudden jump in Roa when the fish’s trajectory crosses the positive x-semi-axis. During steering, the pattern of Roa changes, resulting in a ‘∨’ or ‘∧’ shape. To facilitate subsequent analysis, we define the turning moment as the point where the ‘∨’ or ‘∧’ curve peaks. This moment indicates that the fish’s movement has changed from clockwise to counterclockwise or vice versa. As the U-turn process of the fish is rapid, the individual’s movement direction at the turning moment is approximately perpendicular to the tangential direction of the annular water tank. Additionally, we define the turning moment of a pairwise U-turn process as the intermediate moment between the two fishes’ turning moments.

**Figure 7:**
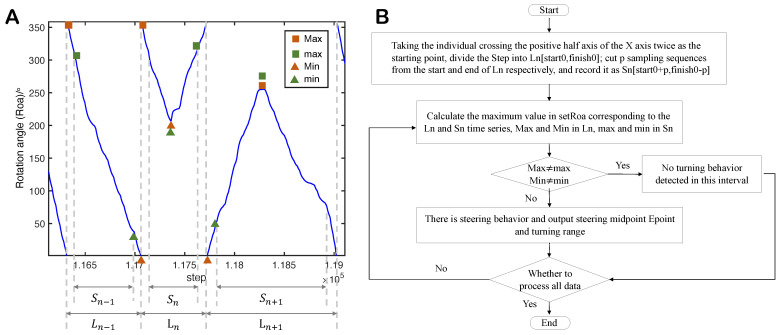
(**A**) Schematic diagram of the principle of single-turn recognition extraction algorithm on position rotation angle (Roa) images. (**B**) Block diagram of a single-turn recognition extraction algorithm based on the position rotation angle.

We design a single-turn identification method based on the Roa. First, we calculate each individual’s position rotation angle dataset (setRoa). Simultaneously, a sampling sequence dataset (Step) is generated to number the sampling sequence of the motion track dataset. Next, we identify the steering feature in setRoa and determine the corresponding turning moment of the U-turn process in Step, outputting the sequence segment of the steering process. A block diagram of the single-turn identification algorithm is depicted in Figure 7. Examples of the algorithm’s recognition results can be found in Table 2. The algorithm process is divided into the following four steps:

1. Data Segmentation: We segment the Step by using the moments when an individual crosses the positive half-axis of the x-axis consecutively as starting and ending points, denoted as Ln[start0,finish0]. Subsequently, we extract subsequences of length *p* from the beginning and end of Ln, and the remaining sequence is denoted as Sn[start0+p,finish0−p]. The length *p* should be greater than half of a single turning time and less than a complete turning time, and in this context, we set p=20.
2. Calculate the maximum values: Find the extreme values in the setRoa data corresponding to the Ln and Sn time series, respectively. The extreme values of Ln are recorded as Max and Min, while the extreme values in Sn are recorded as max and min.
3. Turning judgment: If Max≠max and Min≠min, there is no turning behavior in this Ln segment. However, if Max=max or Min=min, turning behavior exists. The sampling point corresponding to the extremum is the Epoint, representing the turning midpoint of the paired U-turn process, where the individual’s movement direction is perpendicular to the wall.
4. Output Epoint and the turning sequence interval [Epoint−q,Epoint+q]. Here, *q* is used to adjust the length of the output turning interval, and it is determined based on the motion characteristics of the fish.

### 3.3. Formulation of State Parameters

The pairwise movement dataset comprises position data for each fish, and the vector from the center of the circle to the individual position is represented as $\vec{c(t)}$. Given the sampling frequency of 50 Hz, the trajectory between two consecutive sampling points is approximated as a straight line. The fish’s heading is represented by $\vec{heading(t)}=\vec{c(t)}-\vec{c(t-1)}$, while the heading change is denoted by $\vec{dheading(t)}=\vec{heading(t)}-\vec{heading(t-1)}$.

For analytical purposes, the average body length of the fish is denoted as BL, and the movement is treated as that of a rigid body, disregarding any changes in body curvature. The individual’s head and tail positions are approximated based on the position, the direction of movement, and the body length. The position of the fish eye is approximated to the top of the head. The vector from the center of the circle to the individual’s eyesight is expressed as $\vec{e(t)}=\vec{c(t)}+0.5\times BL\times \frac{\bar{heading(t)}}{|\bar{heading(t)}|}$, and the vector from the center of the circle to the individual’s tail is expressed as $\vec{t(t)}=\vec{c(t)}-0.5\times BL\times \frac{\bar{headng(t)}}{|\bar{heading(t)|}}$.

In the context of paired fish movement, the leader in a pair of fish is operationally defined as the individual exhibiting forward movement along the direction of the group’s motion, and the other is the follower. The leader and follower coordinates are denoted as Lc(t) and Fc(t). Vectors $\vec{Lc(t)}$ and $\vec{Fc(t)}$ originate from the circle center and extend to the position of the individual leader and follower, respectively. At time *t*, $\vec{LHeading(t)}$ and $\vec{FHeading(t)}$ indicate the headings of the leader and follower. In contrast, the changes in their respective headings are indicated by $\vec{D_{l}Heading\left(t\right)}$ and $\vec{D_{f}Heading\left(t\right)}$. Additionally, Lspeed(t) and Fspeed(t) represent the instantaneous speeds of the leader and follower at time *t*.

The relative distance between two individuals is denoted as Fdis:

(2) $$Fdis\left(t\right)=\frac{|\vec{Lc(t)}-\vec{Fc(t)}|}{BL}=\frac{dis}{BL}$$

where dis represents the actual distance between two individuals. The minimum relative distance from an individual to two walls is recorded as Wdis:

(3) $$Wdis\left(t\right)=\frac{min\left\{\left(\left|\vec{c(t)}\right|-r\right),\left(R-\left|\vec{c(t)}\right|\right)\right\}}{BL}=\frac{min(w,W)}{BL}$$

where *R* is the outer diameter of the annular water tank, and *r* is the inner diameter. *w* and *W* are the distance from the individual position to the inner and outer walls. $W_{l}dis\left(t\right)$ and $W_{f}dis\left(t\right)$ are the shortest relative distance between the leader and the follower at time *t* and the wall, respectively.

The relative position angle, denoted as Rpa, is the angle between the line connecting the follower’s position to the leader’s position and a reference vector $\vec{FHeading(t)}$. This angle serves to represent the relative orientation of the leader concerning the follower:

(4) $$Rpa\left(t\right)=∠\left(\vec{Lc(t)}-\vec{Fc(t)}\right),\vec{FHeading}>$$

The alignment degree [9], denoted as ald(t), is defined as the sine value of the angle θ formed by vectors $\vec{Heading(t)}$ and $\vec{c(t)}$ representing an individual’s orientation at time *t*. It quantifies the relationship between the individual’s orientation and the wall. This metric is a crucial indicator for assessing the individual’s orientation in a circular pool. The variables Lald(t) and Fald(t) represent the alignment between the leader and the follower.

Los is the angle formed by the projection of the leader within the follower’s field of view:

(5) $$Los\left(t\right)=∠\left(\left(\vec{Le(t)}-\vec{Fe(t)}\right),\left(\vec{Lt(t)}-\vec{Fe(t)}\right)\right)$$

### 3.4. Betweenness Centrality Score

The betweenness centrality score (BCS) is a network node importance index calculated based on the shortest path, representing the dependence of different parameter points in the network. The higher the score, the more influential the node is in the network. Table 3 is the BCS table for the clustering results of the swimming process of the two-fish system.

## 4. Discussion

We investigated the paired U-turn movement of two fish in an annular tank and identified two distinct types of turns: *dominated turn* and *non-dominated turn*. These two turning modes exhibit notable differences in macroscopic movement characteristics, data characteristics, and state composition. In a *dominated turn*, there is a clear leader, and the turning process is short, with a considerable distance between the two individuals. In a *non-dominated turn*, there is no fixed leader, the turning process takes longer, and the distance between the two individuals is shorter. Overall, the *dominated turn* process is characterized by orderly and efficient movement, while the *non-dominated turn* process is complex and intricate. Analyzing the movement state compositions of the two-fish system during the turning process, we found higher motion consistency during the dominated turn process. In contrast, the motion states in the non-dominated turn are more complex, with an attention shift. In addition, we found that wall avoidance is the main factor for turning. When the distance between individuals is appropriate, the movement process is orderly and efficient, making it more conducive to a *dominated turn*. Conversely, when the distance is too short, it triggers a *non-dominated turn*.

Furthermore, the centrality analysis of the clustering network obtained through TICC reveals that the speed of the individual and the relative orientation of the two individuals have an insignificant impact on the motion state of the two-fish system. Burst-gliding behavior is the fundamental action of fish swimming, and there are notable distinctions in the duration and rate of change between its two stages. This could explain the lack of emphasis on movement speed. The frequent speed changes may result in overwhelming information, leading individuals to prioritize their neighbors’ directional and distance information instead.

Time-series clustering analysis treats two fish as a single system and clusters the system’s motion into different states. Analyzing the specificity of combinations between different states and the attributes associated with each state aids in comprehending the characteristics and influencing factors of paired U-turn movements in fish. Our research demonstrates that using time-series clustering to process biological collective-motion data is a practical analytical approach. In our future study, we will apply this method to analyze biological collective behaviors. It is worth noting that the key to this method lies in creating a dataset that can describe the motion states of the system. The system states encompass the motion states of each individual in the system, the relative relationships among individuals, and the relationships between the system and the external environment. In the two-fish system used herein, we utilized ten state parameters to characterize the system’s motion states. Among them, four parameters described the state of each individual, quantifying the individual’s speed and changes in heading. Two parameters captured the relationships between individuals, including the distance between individuals and the leader’s direction relative to the follower. Four parameters described the relationships between the system and the external environment, encompassing the distance between individuals and walls and the movement direction of individuals relative to the walls. As the number of individuals in the system increases, the relationships between individuals become more complex, requiring the construction of additional parameters for description, posing challenges for analysis and computation.

## Figures and Tables

**Table 1:** State composition of two types of U-turns in the early stage.

Dominated Turn		Non-Dominated Turn	
Epoint	State Composition	Epoint	State Composition
97,745	5-2-5	57,789	5-2-4-3-5
104,912	5-2-1-5	65,329	5-3-5-1-5-3-2-4
105,600	5-2-4-2-5	86,581	3-4-2-4-2-4-2
106,120	5-2-1-5	110,763	5-4-5-1-4-5
109,815	5-2-4-5	117,352	4-5-1-2-1-3-5
112,545	5-2-5	181,704	3-5-3-2-5
114,646	5-2-3-5	189,843	1-5-4-5-3

The table displays the early-stage state composition of two types of U-turn. The Epoint represents the pairwise U-turn moment in the dataset.

**Table 2:** The results of using the automatic recognition and extraction method for turning to extract data from two sets of original location data.

Data	exp02H20141127_14h13											
**Epoint**	57,789	65,329	73,042	80,015	81,039	86,580	97,745	104,912	**105,600**	109,815	110,761	112,544
	114,645	117,351	118,241	**119,386**	130,286	139,795	149,954	152,681	168,363	169,677	169,909	170,384
	181,704	186,981	189,842	190,751								
**Data**	**exp02H20141205_16h11**											
**Epoint**	1424	5449	11,609	21,780	24,928	29,477	32,294	35,677	36,081	37,278	56,062	59,098
	65,958	116,047	120,985	125,856	126,710	133,707	134,831	135,466	141,762	152,845	157,638	162,542
	163,199	171,590	172,388	174,741	175,396	178,549	180,197	186,365	188,285	189,065	220,371	226,732
	233,093	239,453	245,814	252,175	258,536	264,896						

The two sets of original position data were extracted using the automatic recognition and extraction method for turning. The data in the table shows the Epoint for each group of turning, and the bold data shows the undetected points. There were a total of **66** single turns and **64** detections. The reason for not being detected is that the turning midpoint is too close to the positive coordinate axis, so the length of Ln is less than 2*p*, resulting in algorithm failure.

**Table 3:** Betweenness centrality score of each state parameter of clustering results.

	Fdis	$W_{l}\mathrm{dis}$	$W_{f}\mathrm{dis}$	$\vec{D_{l}\mathrm{Heading}}$	$\vec{D_{f}\mathrm{Heading}}$	Lspeed	Fspeed	Lald	Fald	Rpa
Cluster 1	0.435444	1.000000	0.060832	0.2545	0.202669	0	0	0.141837	0.275916	0.006207
Cluster 2	0.078841	0.492988	1.000000	0.00373948	0.006232	0	0	0.680274	0.735743	0
Cluster 3	0.324236	0.213974	0.578057	0.402838	0.561135	0.072052	0.025109	0.889738	1.000000	0.0131004
Cluster 4	0.198903	0.823942	1.000000	0.113616	0.10661	0.059092	0.08742	0.286019	0.264392	0.209869
Cluster 5	0.041522	0.415216	0.30358	0.253854	1.000000	0	0.00721	0.047737	0.222775	0

The parameters in the table represent the meanings represented: Fdis is the normalized distance between two individuals. $W_{l}dis$ and $W_{f}dis$ are the shortest relative distance between the leader and the follower and the wall, respectively. $\vec{D_{l}Heading}$ and $\vec{D_{f}Heading}$ indicate the changes in heading for the leader and follower, respectively. Lspeed and Fspeed represent the instantaneous speeds of the leader and follower. Lald(t) and Fald(t) represent the alignment between the leader and the follower. Rpa represents the relative orientation of the leader to the follower. The definitions and calculation methods for each parameter can be found in “Section 3.3”.

## Data Availability

Publicly available datasets were analyzed in this study. Data from ’Social conformity and propagation of information in collective u-turns of fish schools’. This dataset can be found here: https://doi.org/10.5061/dryad.9m6d2 (accessed on 28 March 2018).
